# The mechanism of primary patellar dislocation: Trauma history of 126 patients

**DOI:** 10.3109/17453670903110634

**Published:** 2009-08-01

**Authors:** Risto Nikku, Yrjänä Nietosvaara, Kari Aalto, Pentti E Kallio

**Affiliations:** ^1^Peijas Hospital, Helsinki University Central HospitalPl 900 HUSFinland; ^2^Orto-lääkärit Lääkäriasema Oy, HelsinkiFinland; ^3^Hospital for Children and AdolescentsHUSFinland

## Abstract

**Background and purpose** Several mechanisms are responsible for patellar dislocation. We investigated how the primary pathomechanism relates to patient characteristics and the outcome.

**Methods** 126 patients (81 females) with primary patellar dislocation reported the knee position before the episode, the movement during it, and whether the patella was locked in dislocation. The median age was 20 (9–47) years. The subjective outcome and Kujala, Hughston VAS, and Tegner scores were evaluated after an average of 7 years.

**Results** 102 patients moved to flexion during the dislocation, 98 from a straight start and 4 from a well-bent start. 10 extended the knee from a well-bent start; they were older (mean 25 vs. 19 years) and more often had low trauma energy (5/10 vs. 15/102) and a locked dislocation (10/10 vs. 50/102). 4 had a direct hit to the knee and 1 only rotated it while stretching. 24 of 60 patients with open growth line of the tibial tubercle and 43 of 66 with closed tubercle had locked primary dislocation (p = 0.005). 33% of girls, 52% of boys, 57% of women, and 71% of men had locked primary dislocation. There was no correlation between trauma mechanism and outcome.

**Interpretation** Movement to flexion occurred in 84% of primary patellar dislocations and movement to extension in 8%. Spontaneous patellar relocation is common in skeletally immature girls and locked dislocation is common in skeletally mature men.

## Introduction

A knee cap locked over the lateral femoral condyle is an obvious sign of dislocation, but after spontaneous relocation the diagnosis is more difficult.

The patella is presumed to dislocate at the start of knee flexion, with the quadriceps muscle acting as a lateralizing force (Hughston et al. 1984). Dislocation in a more flexed position, while sitting, has also been described (Maletius et al. 1994). We classified the trauma histories and assessed the association between trauma mechanism, patient characteristics, and outcome in a large series of patients.

## Patients and methods

We prospectively studied 126 knees in 126 patients with primary dislocation of the patella, 81 of whom were females (for inclusion criteria and criteria for diagnosis and treatment, see Nikku et al. 2005). The median age of the patients was 20 (9–47) years and the growth line of the tibial tubercle was still open in 60.

The starting position of the knee (straight or bent), knee movement during the dislocation (flexion or extension), any hit, and the amount of energy were evaluated. The outcome was assessed after a median follow-up time of 7 (6–9) years. This consisted of a four-digit subjective opinion, 2 knee scores (Flandry et al. 1991, Kujala et al. 1993), 1 activity score (Tegner and Lysholm 1985), patellofemoral stability, trust to the knee, and whether there were any later operations (Nikku et al. 2005).

### Statistics

The sample means were compared by the Mann-Whitney rank sum test and the differences in proportions by the chi-square test with continuity correction of Yates (f ≤ 20), and Fisher's exact test (n ≤ 20, f ≤ 4). Significance was set at p = 0.05.

## Results

**Figure 1.**
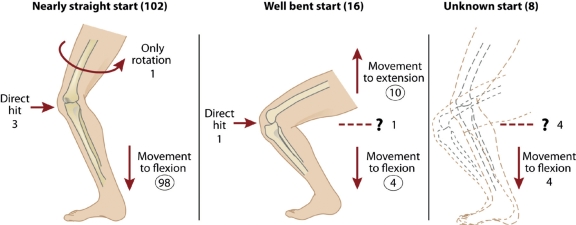
The position of the knee before the primary patellar dislocation and movement during the episode according to the trauma history of 126 patients. Values shown with circles were analyzed further.

The position of the knee before the dislocation had been almost straight in 102 of the 126 patients (Figure 1). 10 patients moving to extension were older (mean 26 years) than the 102 moving to flexion with any start (mean 19 years; p = 0.05) (circled in Figure 1). The former group all had a locked dislocation, as compared to 50 locked dislocations in the 102 patients moving to flexion (p = 0.002). Finally, 5 of the 10 knees moving to extension had low-energy trauma (standing up etc.), as compared to 15 of the 102 knees moving to flexion (p = 0.02). No correlation was found between the three most common trauma mechanisms and outcome (circled in Figure 1).

**Figure 2.**
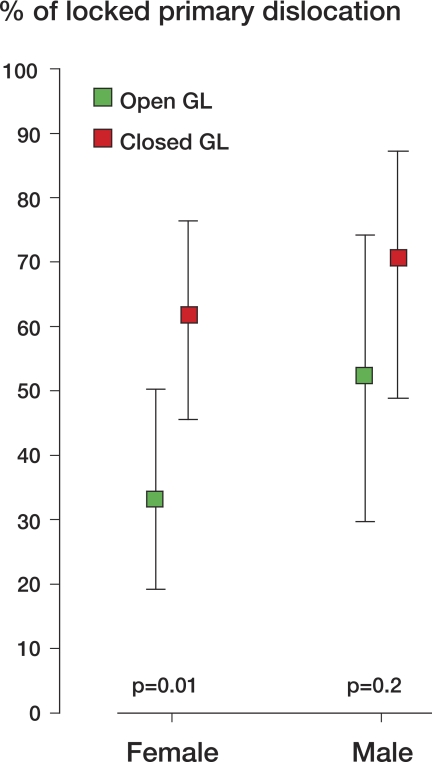
Incidence (with 95% CI) of locked primary patellar dislocation according to sex and skeletal maturity of the tibial tubercle growth line (GL).

Proportionately more of the 66 patients with closed growth line of the tibial tubercle had a locked primary dislocation (43/66 as opposed to 24/60 with open growth line; p = 0.005) (Figure 2). There was no correlation between gender and locking (39 locked/81 females vs. 28 locked/45 men; p = 0.2) or age and locking (mean 21 years for locked vs. 18 years; p = 0.1).

## Discussion

The patient's description of the trauma is usually only indicative of the true trauma mechanism. On rare occasions, the trauma can be visualized by video or film recording. Furthermore, MRI may show bone bruises and allow location of the trauma. In most cases, however, the patient history is still required in order to analyze the details of movement.

In addition to the most common displacement mechanism at the start of flexion, probably at the upper part of the patellofemoral joint, we can describe two other possibilities for the patella to dislocate in a well-flexed knee: (1) an accelerating mechanism (8%), with a squat start and dislocation while extending the knee; (2) a decelerating mechanism (3%) with a well-flexed start (70–90°) and dislocation during further flexion. Standing up from a seat, taking off to a jump, and lifting from a bent position are examples of accelerating movement from a bent start. We presume that the displacement takes place during the movement and not after it. The painful dislocation may stop the active muscle work so that the patient falls, which occurred in 9 of our 126 patients.

An osteochondral fracture may destroy the congruence of bone. This is why our study consisted of selected patients without marked osteochondral fractures to cause instability.

The femoral facies patellaris ends distally at two oblique crests: the medial and lateral linea terminalis of femur. The crest is followed by a transverse groove, sulcus terminalis, which is deeper on the lateral condyle (Scheller and Mårtenson 1974). In a well flexed knee, the quadriceps tendon has set on the femur at 70° of flexion, reducing further pressure to the patella (Hehne 1990). At this position, the quadriceps tendon may be displaced from the bottom of the sulcus onto the lateral crest of femur. This more anterior, higher tendofemoral contact can decompress the patellar stability near the transverse sulcus terminalis.

Primary dislocation presenting in a skeletally immature knee is also more unstable at the displaced position (Figure 2). Spontaneous relocation often takes place in girls with open growth line of tibial tubercle, and locking often takes place in skeletally mature men.
